# Oral Health—Head and Neck Cancers: Addressing Confounding Through Negative Control and Quantitative Bias Analyses

**DOI:** 10.1111/cdoe.70046

**Published:** 2025-12-17

**Authors:** P. K. Elango, B. Nicolau, N. Farsi, A. V. Grant, M. C. Rousseau, S. Madathil

**Affiliations:** ^1^ Faculty of Dental Medicine and Oral Health Sciences McGill University Montreal Quebec Canada; ^2^ Department of Preventive Dental Sciences King Abdul Aziz University Jeddah Saudi Arabia; ^3^ Department of Anaesthesia, Faculty of Medicine and Health Sciences, Alan Edwards Centre for Research on Pain McGill University Montreal Quebec Canada; ^4^ Epidemiology and Biostatistics Unit, Centre Armand‐Frappier Santé Biotechnologie Institut National de la Recherche Scientifique Laval Quebec Canada; ^5^ School of Public Health Université de Montréal Montreal Quebec Canada; ^6^ Innovation Hub Centre de Recherche du Centre Hospitalier de l'Université de Montréal (CRCHUM) Montréal Quebec Canada

**Keywords:** bias analysis, epidemiology, head and neck cancers, negative controls, oral cancers, oropharyngeal cancers

## Abstract

**Objectives:**

While there are plausible biological explanations for the association between oral health and head and neck cancers (HNC), existing studies have yielded conflicting results. A key concern is that these associations are influenced by mediators, unmeasured risk factors, and biases. To address this, a negative control exposure was used to evaluate whether the associations between oral health and HNC risk could be attributed to unmeasured confounding. Additionally, quantitative bias analysis (QBA) was performed to estimate the extent of non‐differential misclassification of exposure.

**Methods:**

The HeNCe study, a hospital‐based case–control study, recruited incident HNC cases (*n* = 389) frequency matched to controls (*n* = 429) by sex and age (within 5 years) from four major referral hospitals in Montreal, Canada. In‐person interviews collected information on life course exposures. Unconditional logistic regression estimated the odds ratios (OR) and 95% confidence intervals (CI) for the associations between oral health indicators and HNC, controlling for confounders identified using directed acyclic graphs (DAG). Sexually transmitted diseases (STD) were used as a negative control exposure to test for unmeasured confounding in the associations. QBA, using predetermined bias parameters from previous studies, estimated the magnitude and direction of exposure misclassification bias.

**Results:**

Complete denture use and having more than nine missing teeth were associated with an increased HNC risk [OR = 1.33, 95% CI (0.93–1.90) & OR = 1.31, 95% CI (0.93–1.83)], respectively. Similar results were obtained when stratified by HNC subsite. Negative control analysis yielded a null finding, indicating no significant bias due to unmeasured confounders. Bias‐corrected estimates of the association between oral health indicators and HNC risk moved further from the null.

**Conclusion:**

Negative control exposure analysis indicated that unmeasured confounding did not affect the association between oral health and HNC risk. QBA yielded corrected estimates of increased magnitude, suggesting that the crude associations may have been underestimated.

**Abbreviations**
aOR: adjusted odds ratio
CI: confidence interval
DAG: directed acyclic graph
HeNCe: head and neck cancer life study
HIV: human immunodeficiency virus
HNC: head and neck cancers
HNSCC: head and neck squamous cell carcinomas
HPV: human papilloma virus
OPC: oropharyngeal cancers
OR: odds ratio
QBA: quantitative bias analysis
STD: sexually transmitted diseases

## 1 Introduction

Head and neck cancers (HNC) are malignant neoplasms primarily of epithelial origin, affecting the oral cavity, pharynx, nasal cavity, paranasal sinuses, and larynx [1]. They are the seventh most common cancer worldwide, with about 660 000 new cases and 325 000 deaths annually [2]. HNC is a complex, multifactorial disease with established risk factors, such as tobacco use, alcohol consumption, and human papillomavirus (HPV) infection. Other HNC risk factors include poor oral health, diet, obesity, and occupational exposure [3, 4, 5].

With advances in understanding the role of the oral microbiome and chronic inflammation in carcinogenesis, oral health has recently received increasing attention in HNC aetiology. Despite plausible biological mechanisms that may promote carcinogenesis, such as persistent low‐grade inflammation, alterations in the oral microbiome, and impaired nutritional status [6, 7], these associations have not been consistently reflected in epidemiological studies. While studies have reported positive associations between oral health indicators (e.g., periodontal disease, tooth loss, denture use, and mouthwash use) and HNC risk [8, 9], other studies have not observed such associations [10, 11]. Arguably, these are complex associations and may be spurious, arising from unmeasured confounders, mediators, and biases in observational studies [12]. To address these uncertainties, we applied epidemiological tools that are not often used in oral epidemiology to evaluate whether the observed associations could be explained by unmeasured confounding and systematic biases.

Negative control analysis can detect potential unmeasured confounding, selection bias, and measurement error [13]. This approach introduces an exposure that is not expected to have a causal relationship with the outcome; an observed association would suggest residual bias, whereas a null finding would strengthen the validity of the main association [13, 14]. Misclassification bias, arising when exposures or outcomes are inaccurately recorded, can be assessed using quantitative bias analysis (QBA). Non‐differential misclassification typically biases results toward the null, while differential misclassification can bias results in either direction [15]. QBA provides a structured framework to quantify the potential impact of such errors by specifying plausible sensitivity and specificity values and simulating their effect on the study estimates [16].

Accordingly, the study aimed to assess the potential impact of unmeasured confounding and misclassification bias on the association between oral health and HNC by applying negative control analysis and QBA in a hospital‐based case–control dataset. Through these techniques, the robustness of the observed associations was evaluated, and bias‐corrected estimates were obtained.

## 2 Methods

Data came from the HeNCe Life study, a hospital‐based case–control study conducted in four main referral hospitals in Montreal, Canada, between September 2005 and November 2013. Informed consent was obtained from each participant, and approval was secured from the Ethics Review Authorities at all participating institutions. The study, previously described [17], included 389 consecutive incident histologically confirmed head and neck squamous cell carcinoma cases (oral cavity, oropharynx, and larynx), identified using the International Classification of Diseases (ICD‐10) [18]. Cancers of the lip, salivary glands, nasopharynx, and oesophagus were excluded due to etiological and histological differences. Controls (*n* = 429) frequency matched to the cases, by sex and age (5‐year brackets), were randomly selected from several outpatient clinics in the same hospitals during the same period. To further ensure that cases and controls were drawn from the same study base, controls were restricted to outpatient clinics treating conditions unrelated to tobacco and alcohol use, with no single diagnostic group contributing more than 20% of the sample, and all participants (cases and controls) residing within 50 km of the hospital catchment area.

Face‐to‐face interviews using a questionnaire with a life grid [19] collected information on an array of life course exposures, including sociodemographic characteristics (e.g., age, education, socioeconomic position) and behavioural factors (e.g., tobacco smoking, alcohol consumption). Oral rinse and oral brush protocols were used to collect oral trans‐epithelial cells from the oral cavity. Samples were stored in PreservCyt buffer solution (Hologic, Bedford, MA) at four degrees Celsius until testing. HPV DNA detection and genotyping were performed using Linear Array (Roche Molecular Diagnostics, Pleasanton, CA) [20].

### 2.1 Main Exposure: Oral Health Indicators

Clinical oral health measures were not recorded in the HeNCe study. Self‐reported information was collected on several oral health indicators, including complete denture use, ever‐use of mouthwash, and the number of missing teeth, before any treatment for HNC [21]. The first two variables were binary (yes/no). Using an ordinal scale (none, one to five, six to fifteen, sixteen to twenty, twenty‐one to thirty, and more than 30), participants selected the option that corresponded to the number of permanent teeth lost in each specific period (childhood, early adulthood, and late adulthood). To calculate the lifetime number of missing teeth, the midpoint of each category for all three time periods was selected and summed. For example, if a participant reported losing one to five teeth in childhood, none in early adulthood, and six to fifteen in late adulthood, the total number was calculated as 3 + 0 + 10 = 13. The resulting continuous variable was then categorised into nine or fewer and more than nine missing teeth, using the 50th percentile among the control group as the cut‐off point.

### 2.2 Negative Control Exposure

**FIGURE 1:**
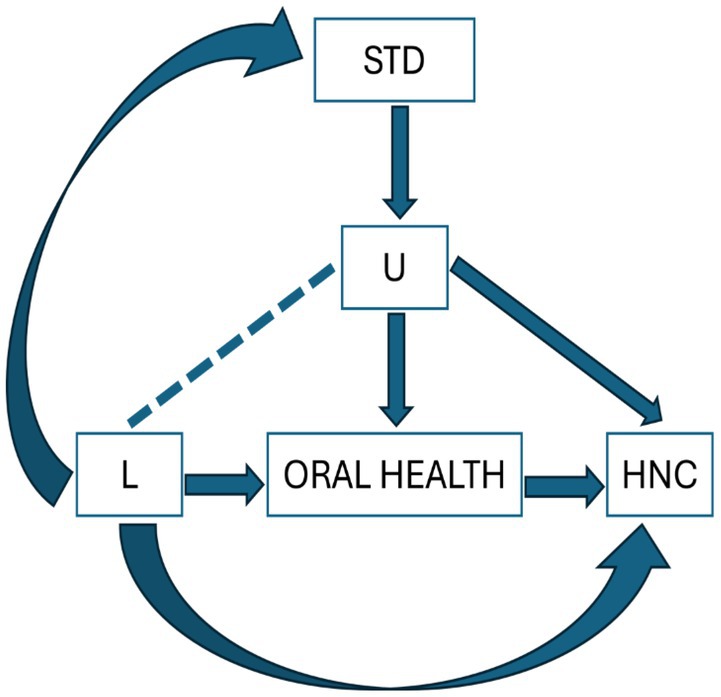
A diagrammatic illustration of the relationship between the exposure (oral health), outcome (HNC), negative control exposure (STD), measured confounders (L; alcohol, smoking, HPV), unmeasured confounders (U).

A negative control exposure variable must not causally affect the outcome but must have a similar confounding structure to the exposure of interest, that is, the confounding structure should be the same between the negative control exposure and outcome as it is with the exposure of interest and the outcome Figure 1 [14]. Our negative control exposure was the self‐reported lifetime presence of any sexually transmitted diseases (STDs) (e.g., syphilis, gonorrhoea, chlamydia, and herpes), as they have not been linked to HNC risk [22, 23, 24]. Importantly, the STD variable excluded HPV infection to avoid introducing bias. HPV was accounted for as a separate confounder in the models. Participants with an immunocompromised status, such as HIV, were also excluded from the HeNCe study based on predefined criteria.

### 2.3 Quantitative Bias Analysis

Self‐reported data are prone to misclassification and recall biases. QBA was performed to determine the extent of misclassification bias, which was assumed to be non‐differential. The objective was to provide a reasonable estimate of the potential effects of misclassification bias on the observed results. Assuming a single deterministic value for bias parameters is unrealistic [25]. For QBA, a probability distribution of the bias parameters was assumed; in this case, a beta distribution was used [26]. The priors or the probability distribution parameters (sensitivity and specificity) of each oral health variable were determined by expert opinion and previous validation studies [27, 28, 29, 30, 31, 32]. The mouthwash use variable was excluded from this analysis since it could not be clinically determined, and no gold standard exists for its measurement. For the other variables, predetermined priors were obtained by the best assumption, considering estimates from existing validation studies of self‐reported oral health measures, as described in Table S1 [27, 28, 29, 30, 31, 32]. For example, it was assumed that self‐reported missing teeth were non‐differentially misclassified with a probability between 75 and 85, 95% of the time (95% CI), so a sensitivity prior of 80 [75–85] was used for missing teeth. The assumptions need not be perfect, but were expected to be reasonable and well‐described to be informative [16]. The extent of bias in the associations was estimated and the estimates were corrected under these assumed priors.

### 2.4 Statistical Analysis

Unconditional multivariable logistic regression was used to estimate the odds ratios and corresponding 95% confidence intervals for the associations between oral health indicators and HNC risk (Table 2). All models assessing HNC risk overall and stratified by subsite (oropharyngeal, laryngeal, and oral cancer) were adjusted for the minimal sufficient set of potential confounders identified in a DAG for the association between oral health and HNC risk [33] (Data S1).

Subsequent analyses involved conducting negative control exposure analysis to test the association between STDs and HNC. This was done by substituting oral health with STDs in both crude and adjusted models and running the models to obtain the estimates. These models were executed similarly to those assessing oral health and HNC risk and adjusted for the same set of confounders. An observed null finding would indicate the absence of detectable unmeasured confounder bias. Conversely, an association between STDs and HNC risk would suggest the presence of bias in the original association.

For the QBA, the optimal point estimates were determined for each variable's sensitivity and specificity, based on expert opinion. To obtain a conservative range for the priors, five points were added and subtracted from each prior's point estimate to define confidence intervals. These values were then entered into the model as sensitivity and specificity distribution parameters to assess non‐differential misclassification bias. 20 000 simulations were conducted using the ‘episensr’ package in Rstudio 2022 [34].

## 3 Results

The mean [standard deviation] age of cases and controls was 61.7 years [10.4] and 61.1 years [10.9], respectively, with most of the cases being males [74%]. Oropharyngeal cancer was the most common [188], followed by laryngeal [128] and oral cavity cancers [73]. Controls had 1.8 more years of education than cases. More cases were smokers, consumed alcohol, and had high‐risk HPV exposures compared to controls. Approximately 60% of cases and 45% of controls reported having more than nine missing teeth. Complete denture use was also more prevalent among cases (46%) than controls (31%) (Table 1).

**TABLE 1:** Selected characteristics of the study population (*n* = 818).

Variables	Cases *n* = 389	Controls *n* = 429
Age mean [SD]	61.7 years [10.4]	61.1 years [10.9]
Sex		
Male	288 (74.0)	297 (69.2)
Female	101 (26.0)	132 (30.8)
Tumour site		
Oropharynx	188 (48.3)	—
Larynx	128 (32.9)	—
Oral cavity	73 (18.7)	—
Education years, mean (SD)	12.1 [3.88]	13.9 [4.37]
Lifetime smoking, mean (SD)	40.5 [45.6]	24.2 [38.5]
Lifetime alcohol consumption, mean (SD)	659.8 L[1390.3]	366.8 L[828]
HPV		
HR‐HPV individuals	150 (38.5)	50 (11.65)
LR‐HPV individuals	239 (61.5)	379 (88.35)
Missing teeth		
Nine or fewer	157 (40.3)	237 (55.2)
More than nine	232 (59.7)	192 (44.8)
Complete denture use		
No	209 (53.7)	294 (68.5)
Yes	178 (45.7)	135 (31.4)
Mouthwash use		
No	182 (46.7)	209 (48.7)
Yes	204 (52.4)	218 (50.8)
Negative control exposure‐STD		
Presence of STD	64 (16.4)	73 (17.0)
Absence of STD	322 (82.7)	354 (82.5)

*Note:* HPV status categorised as high‐risk HPV and low risk HPV groups, HPV negative individuals included in low‐risk HPV group. HPV status categorised as high‐risk HPV and low risk HPV groups, HPV negative individuals included in low‐risk HPV group.

Abbreviations: HPV, human papillomavirus; HR‐HPV, high‐risk human papillomavirus; LR‐HPV, low‐risk human papillomavirus; SD, standard deviation; STD, sexually‐transmitted disease.

Compared to individuals who did not wear dentures and had nine or fewer missing teeth, the adjusted odds ratio (aOR) for complete denture use [aOR 1.33 (95% CI: 0.93–1.90)] and for more than nine missing teeth [aOR 1.31 (0.93–1.83)] suggested a 33% and 31% increase in the odds of HNC, respectively, although the CIs were wide (Table 2). The aORs by anatomical subsite were also in the positive direction; oropharyngeal cancer [complete denture: aOR = 1.43 (95% CI: 0.89–2.28); missing teeth: aOR 1.34 (95% CI: 0.86–2.08)] and laryngeal cancer [complete denture: aOR = 1.48 (95% CI: 0.92–2.38); missing teeth: aOR = 1.50 (95% CI: 0.93–2.42)]. The adjusted estimates between mouthwash use and HNC risk yielded values close to null, indicating no association. None of the oral health indicators being examined displayed any apparent associations with oral cancers, which could be potentially due to inadequate subsample size.

**TABLE 2:** Associations between indicators of oral health and HNC overall and stratified by anatomical subsite.

Oral health indicator	Controls *n* = 429	HNC *n* = 389	OR (95% CI)	Oropharyngeal *n* = 188	OR (95% CI)	Larynx *n* = 128	OR (95% CI)	Oral *n* = 73	OR (95% CI)
Complete denture									
No	294	209	1	110	1	59	1	40	1
Yes	135	178	1.33 (0.93–1.90)	78	1.43 (0.89–2.28)	68	1.48 (0.92–2.38)	32	1.01 (0.56–1.83)
Mouthwash use									
No	209	186	1	86	1	66	1	30	1
Yes	218	204	0.99 (0.96–1.01)	101	1.00 (0.98–1.03)	61	0.84 (0.56–1.26)	42	0.97 (0.88–1.71)
Missing teeth									
Nine or fewer	237	157	1	83	1	42	1	32	1
More than nine	192	232	1.31 (0.93–1.83)	105	1.34 (0.86–2.08)	86	1.50 (0.93–2.42)	41	0.99 (0.55–2.0)

*Note:* All models adjusted for age (continuous), sex, education (continuous), lifetime smoking (pack‐years), lifetime alcohol consumption (ethanol litre), HPV status categorised as high‐risk HPV and low risk HPV groups, HPV negative individuals included in low risk HPV group.

Abbreviations: CI, confidence intervals; HNC, head and neck cancers; HPV, human papillomavirus; HR‐HPV, high‐risk human papillomavirus; LR‐HPV, low‐risk human papillomavirus; OR, odds ratio; STD, sexually‐transmitted disease.

Table 3 displays the results of the negative control exposure analysis. Compared to those without STD, the aOR for STDs presence were close to the null value: overall HNC [aOR = 0.99 (95% CI: 0.96–1.01)], oropharyngeal cancer [aOR = 1.00 (95% CI: 0.97–1.03)] and for laryngeal cancer [aOR = 0.87 (95% CI: 0.46–1.03)], indicating no significant detectable bias due to unmeasured confounders.

**TABLE 3:** Associations between STDs and HNC risk factors overall and stratifying by anatomical site: Negative control exposure analysis.

	Cases	Controls	Univariate analysis	Multivariate analysis
			OR (95% CI)	OR (95% CI)
HNC overall	389	429	1.00 (0.98–1.02)	0.99 (0.96–1.01)
Oropharyngeal cancers	188	429	1.00 (0.97–1.02)	1.00 (0.97–1.03)
Laryngeal cancers	128	429	1.00 (0.97–1.02)	0.87 (0.46–1.03)
Oral cancers	73	429	1.01 (0.97–1.03)	0.80 (0.34–1.04)

*Note:* All Multivariate models for the negative control association models were adjusted for age (continuous), sex, education (continuous), lifetime smoking (pack‐years), lifetime alcohol consumption (ethanol litre), HPV status categorised as high and low‐risk groups.

Table 4 represents the bias‐corrected estimates of the oral health indicators, accounting for misclassification bias and random error. The OR for complete denture use increased substantially from 1.83 to 2.97 (95% CI: 2.48–4.30) after accounting for misclassification bias with the selected priors (Se‐ 90%, Sp‐ 80%), indicating previous underestimation. This estimate was further adjusted to 3.01 (95% CI: 2.12–4.72) with the addition of random error correction, indicating a stronger effect. Similarly, for the missing teeth variable, the crude OR of 1.82 (95% CI: 1.38–2.40) reflected a positive association. Bias correction with predetermined priors (Se‐ 80%, Sp‐ 90%) increased the OR to 2.47 (95% CI: 2.25–2.85), indicating a stronger association than initially estimated. When random error was corrected, the OR became 2.48 (95% CI: 1.85–3.37), maintaining the strength of the association.

**TABLE 4:** Systematic and random error corrected estimates from quantitative bias analysis for associations between selected oral health indicators and HNC.

Oral health indicator	Sample size (n)	OR crude (95% CI)	OR sys error corrected (95% CI)	OR Sys err & random error corrected (95% CI)
Complete denture				
Se‐ 90 Sp‐ 80	818	1.83 (1.38–2.44)	2.97 (2.48–4.30)	3.01 (2.12–4.72)
Missing teeth				
Se‐ 80 Sp‐ 90	818	1.82 (1.38–2.40)	2.47 (2.25–2.85)	2.48 (1.85–3.37)

*Note:* All models were univariate crude models which yielded corrected crude estimates of association for the selected sensitivity and specificity priors.

Abbreviations: Se, sensitivity; Sp, specificity.

## 4 Discussion

Despite plausible biological mechanisms, the associations between oral health and HNC remain uncertain and highly contested. This uncertainty may partly explain the contradictory findings in the literature, where both positive associations [7, 8, 9, 22, 35, 36, 37, 38], and null results have been reported [10, 11, 39, 40]. As chronic diseases, both oral conditions and HNC have complex aetiologies and share several common risk factors, making it difficult to disentangle true associations from those that may be spurious, arising from unmeasured confounders, mediators, and biases. Capitalising on a large case–control study of HNC, this study advances the discussion by applying methodological tools rarely used in oral epidemiology, namely negative control analyses and QBA, to evaluate the extent to which such biases may account for the observed associations. The findings are in agreement with the previous literature supporting a positive association [7, 8, 9, 37]. Complete denture use and having more than nine missing teeth were associated with an increased risk of HNC overall, as well as oropharyngeal and laryngeal cancers. Notably, the point estimates observed in this study were strong, although their precision was limited by wide confidence intervals. Interestingly, results from the negative control exposure analysis produced null findings, suggesting that the observed association between complete denture use and a higher number of missing teeth and the risk of overall HNC and subtypes were unlikely to be explained by unmeasured confounding.

Possible explanations for these associations could be through the inflammatory pathway and immune dysregulation leading to carcinogenesis [6, 7]. The QBA revealed stronger associations between oral health and HNC risk after accounting for misclassification bias and random errors. These corrected estimates suggest that non‐differential exposure misclassification likely attenuated the crude and adjusted estimates toward the null, thereby explaining stronger associations after correction. Together, these findings highlight the need for such validation methods and bias‐adjustment techniques to obtain more accurate estimates in epidemiological studies, rather than only acknowledging them as study limitations.

This work has several limitations. First, the hospital case–control design measures outcome and exposure simultaneously, which precludes conclusions about temporality—that is, whether poor oral health (e.g., missing teeth or denture use) preceded the development of HNC or instead resulted from early disease processes. Second, oral health indicators were self‐reported and therefore prone to information bias [41]. Nonetheless, several factors mitigate this concern. Self‐reported oral health has demonstrated good agreement with clinical measures [27, 28, 29, 30, 31, 32, 42], and exposures such as missing teeth and use of dentures are relatively stable over time, limiting recall bias. Moreover, lack of public awareness of the role of oral health in HNC risk at the time of data collection reduced the likelihood of differential misclassification bias. A third limitation relates to QBA, which relies on parameters derived from published studies and may therefore be imprecise. Moreover, despite the use of QBA, additional sources of bias could still distort the estimates.

The HeNCe study also has important strengths. It is one of the largest case–control studies of HNC in Canada, with extensive information on life course exposures. The study interviews were conducted using a structured questionnaire and a life‐grid tool, which reduces recall bias [19]. In addition, several quality control procedures were implemented, including re‐interviews to assess the reliability of responses and interviewing participants' siblings of similar age to validate certain information. Another strength is the incorporation of negative control analyses and QBA. Although these methods are not routinely applied in oral epidemiology, their use adds methodological rigor by formally addressing potential sources of bias and strengthening the credibility of the findings.

In conclusion, the study showed no evidence of unmeasured confounding from the negative control analysis, while QBA indicated that the true relationship between oral health and HNC may be stronger than the crude estimates suggest. Further, longitudinal studies are needed to replicate these results. From a public health perspective, the findings support greater recognition of oral health as part of comprehensive approaches to disease prevention.

## Funding

This work was supported by the Canadian Institutes of Health Research (MOP‐69062, MOP‐20100); Ministère de l’Économie, de l’Innovation et de l’Énergie du Québec (MEIE), de l'Innovation et de l'Exportation du Québec: Programme de Soutien à la Recherche (PSR), volet: Soutien à des Initiatives Internationales de Recherche et d'Innovation (SIIRI).

## Conflicts of Interest

The authors declare no conflicts of interest.

## Supporting information


**Figure S1:** Directed acyclic graph used to identify sufficient set of potential confounders to adjust for in the models.

**Table S1:** Validation Studies of self‐reported oral health measures.

## Data Availability

The data that support the findings of this study are available from the corresponding author upon reasonable request.
