# Supplementary figures and images for: Oral Health—Head and Neck Cancers: Addressing Confounding Through Negative Control and Quantitative Bias Analyses
Source: Community Dent Oral Epidemiol. 2025 Dec 17;54(3):347–53. doi: 10.1111/cdoe.70046 (PMC13146185; doi:10.1111/cdoe.70046)

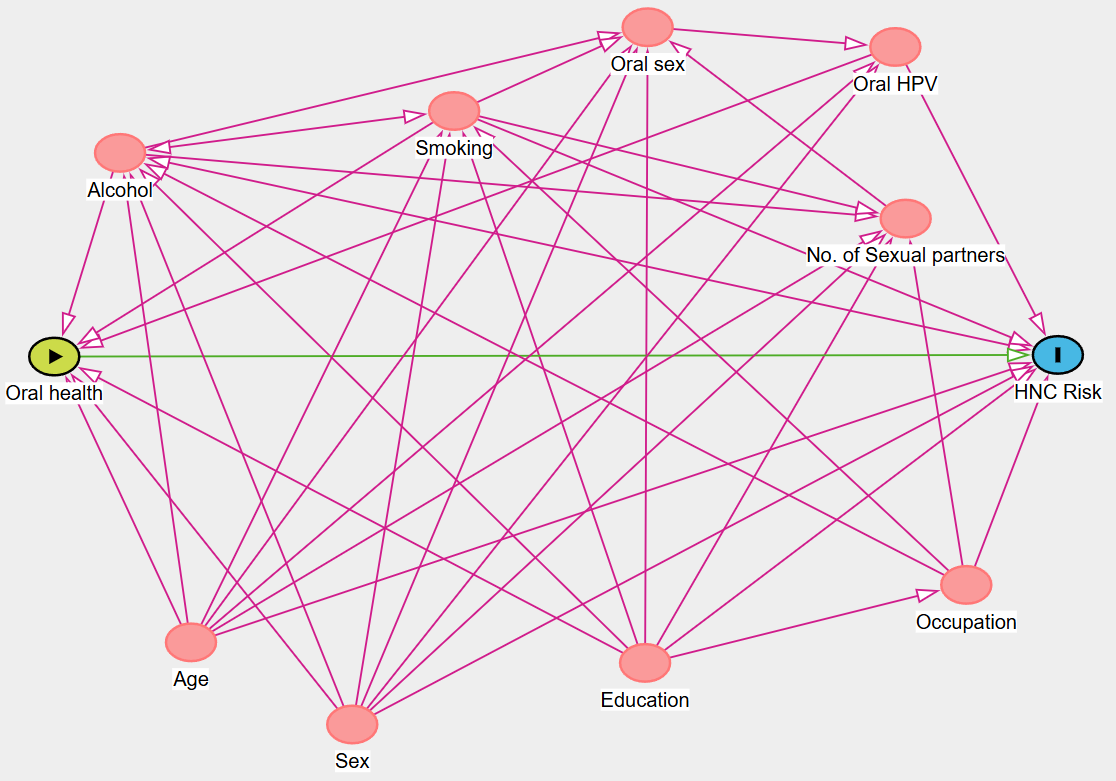

Supplement: Supplementary file 1 — Figure S1: Directed acyclic graph used to identify sufficient set of potential confounders to adjust for in the models. [file CDOE-54-347-s002.tiff]
