# Supplementary material for: Oral Health—Head and Neck Cancers: Addressing Confounding Through Negative Control and Quantitative Bias Analyses
Source: Community Dent Oral Epidemiol. 2025 Dec 17;54(3):347–53. doi: 10.1111/cdoe.70046 (PMC13146185; doi:10.1111/cdoe.70046)
Supplement: Supplementary file 2 — Table S1: Validation Studies of self‐reported oral health measures. [file CDOE-54-347-s001.docx]

**SUPPLEMENTAL MATERIAL FOR NEGATIVE CONTROL MANUSCRIPT**

**Supplemental Table 1 Validation Studies of self-reported oral health measures**

| **Oral health variable** | **Study** | **Sensitivity (Se) value**  **(95%CI)** | **Specificity (Sp) value (95%CI)** |
| --- | --- | --- | --- |
|  |  |  |  |
| **Complete denture** | Arenas-Márquez et al 2019 | 99.3 (97.9-99.8) | 84.4 (79.7-89.3) |
|  |  |  |  |
|  | Ramos et al 2014 | 100% | 100% |
|  |  |  |  |
|  | Pitiphat et al 2002  Selected priors | 100%  90 (85-95) | 93%  80 (75-85) |
|  |  |  |  |
| **Missing teeth** | Balappanavar et al 2011 | 83.8% | 83.3% |
|  |  |  |  |
|  | Sekundo et al 2019 | 93.4 (84.1-98.2) | 92 (85.8-96.1) |
|  |  |  |  |
|  | Ramos et al 2014 | 88-91% | 97% |
|  |  |  |  |
|  | Pinelli et al 2007  **Selected priors** | 96.7%  80 (75-85) | 94.12%  90 (85-95) |
|  |  |  |  |

**Supplemental Material Fig 1 Directed Acyclic Graph**

**Figure tag-**

**Supplemental material Fig**-1 Directed acyclic graph used to identify sufficient set of potential confounders to adjust for in the models
